# (*Z*)-3-Ferrocenyl-2-phenyl­acrylonitrile

**DOI:** 10.1107/S1600536808014517

**Published:** 2008-05-17

**Authors:** Lu-Yang Cao, Heng-Yun Ye

**Affiliations:** a Ordered Matter Science Research Center, College of Chemistry and Chemical Engineering, Southeast University, Nanjing 210096, People’s Republic of China.

## Abstract

In the structure of the title compound, [Fe(C_5_H_5_)(C_14_H_10_N)], the unsubstituted cyclo­penta­diene (Cp) ring is disordered over two positions, with site-occupancy factors 0.76 (2) and 0.24 (2). The dihedral angles between the substituted Cp ring and the major and the minor components of the disordered ring are 0.9 (5) and 6(2)°, repectively. The plane of the acrylonitrile unit makes dihedral angles of 6.1 (18) and 6.5 (4)° with the substituted Cp ring and the phenyl ring planes, respectively.

## Related literature

For background to the chemistry of ferrocene, see: Long (1995 ▶); Roberto *et al.* (2000 ▶); Togni & Hayashi (1995 ▶). For the stuctures of ferrocene derivatives, see: Base *et al.* (2002 ▶); Hess *et al.* (1999 ▶). For bond distances in the acrylonitrile unit, see: Allen *et al.* (1987 ▶).
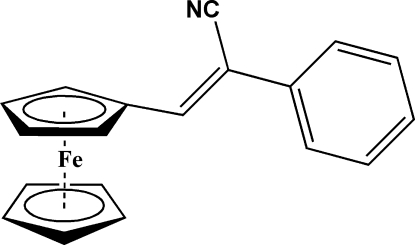


## Experimental

### 

#### Crystal data

[Fe(C_5_H_5_)(C_14_H_10_N)]
*M* *_r_* = 313.17
Monoclinic, 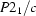

*a* = 6.8255 (14) Å
*b* = 11.795 (2) Å
*c* = 19.939 (4) Å
β = 106.786 (16)°
*V* = 1536.8 (5) Å^3^
*Z* = 4
Mo *K*α radiation
μ = 0.97 mm^−1^
*T* = 293 (2) K
0.18 × 0.06 × 0.05 mm

#### Data collection

Rigaku, SCXmini diffractometer
Absorption correction: multi-scan (*CrystalClear*; Rigaku, 2005 ▶) *T*_min_ = 0.892, *T*_max_ = 1.00 (expected range = 0.850–0.953)
15093 measured reflections
3523 independent reflections
2520 reflections with *I* > 2σ(*I*)
*R*_int_ = 0.050

#### Refinement

*R*[*F* ^2^ > 2σ(*F* ^2^)] = 0.052
*wR*(*F* ^2^) = 0.126
*S* = 1.07
3523 reflections
206 parameters
30 restraints
H-atom parameters constrained
Δρ_max_ = 0.22 e Å^−3^
Δρ_min_ = −0.40 e Å^−3^

### 

Data collection: *CrystalClear* (Rigaku, 2005 ▶); cell refinement: *CrystalClear*; data reduction: *CrystalClear*; program(s) used to solve structure: *SHELXS97* (Sheldrick, 2008 ▶); program(s) used to refine structure: *SHELXL97* (Sheldrick, 2008 ▶); molecular graphics: *SHELXTL* (Sheldrick, 2008 ▶); software used to prepare material for publication: *SHELXL97*.

## Supplementary Material

Crystal structure: contains datablocks I, global. DOI: 10.1107/S1600536808014517/sj2497sup1.cif

Structure factors: contains datablocks I. DOI: 10.1107/S1600536808014517/sj2497Isup2.hkl

Additional supplementary materials:  crystallographic information; 3D view; checkCIF report

## Figures and Tables

**Fig. 1.**
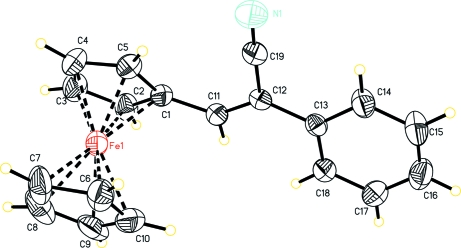
The structure of I showing the atom numbering scheme with displacement ellipsoids drawn at the 30% probability level. For clarity only atoms of the major disorder component of the unsubstituted cyclopentadiene ring are included.

## supplementary crystallographic information

### Comment

The chemistry of ferrocene has received much attention because of its applications in many fields, such as in catalysis, organic or organometallic synthesis and materials (Togni, *et al.*(1995)). The use of ferrocene and its derivatives as non-linear optical(NLO) materials has also been reported (Long, 1995; Roberto *et al.*,  2000). As part of our on going studies of the chemistry of ferrocene, we present here the structure of the title compound, (*Z*)-2-Phenyl-3-(ferrocenyl)acrylonitrile (I).

In I, the unsubstituted cyclopentadienyl (Cp) ring is disordered over two positions, the site occupancy factors are 0.76 (2) and 0.24 (2). The major disorder component is nearly parallel to the substituted Cp ring with a dihedral angle of 0.9 (5)° , and the rings are in an eclipsed configuration with the torsion angle C1-Cg1-Cg2-C10, -5.918 (3)°. The minor component of the disordered Cp ring is tilted slightly, making a dihedral of 6(2)° with C1···C5, and the rings are staggered, C1-Cg1-Cg2'-C9' 44.692 (4)° (Cg(1) denotes the centroid of the C1···C5 Cp ring, Cg(2) and Cg(2)' denote the centroids of the C6···C10 and C6'···C10' rings respectively). Fe-C distances to the substituted Cp ring vary from 2.030 (3) to 2.039 (3)Å, and are in the normal ranges (Hess *et al.*,  1999; Base *et al.*,  2002). Those to the unsubstituted Cp ring cover a wider range from 1.92 (3) to 2.084 (7) Å. The iron-ring centroid distances are Fe–Cg(1), 1.6378 (16) Å, Fe-Cg(2) 1.674 (3) Å  and  Fe–Cg(2)' 1.594 (13) Å. Within the acrylonitrile unit, bond angles and the C11═C12 double bond distance, 1.345 (3) Å) and C19≡N1 distances, 1.142 (4) Å, are normal (Allen *et al.* (1987). The plane of acrylonitrile unit makes dihedral angles of 6.13 ( 1.81 )° and 6.52 ( 0.39 )° with the C1···C5 ring and the C13···C18 phenyl ring plane, respectively.

### Experimental

To a mixture of ferrocenecarboxaldehyde and 2-phenyletonitrile in CH_2_Cl_2_ was added pyrrolidine and the mixture was heated to reflux temperature for 3 h. The reaction was cooled to room temperature and solvent removed. The crude product was purified by column chromatography on silica gel using ethyl acetate-petroleum ether (v:v, 1:3) as eluent to collect the main yellow band. Red crystals  suitable for X-ray analysis were obtained by slow evaporation of a saturated solution of ethyl acetate-petroleum ether (v:v,1:3). BAND YELLOW, CRYSTALS RED. IS THIS CORRECT??

### Refinement

All H-atoms were positioned geometrically and refined using a riding model with d(C-H) = 0.93Å,  U_iso_=1.2U_eq_ (C). The unsubstituted Cp ring is disordered over two positions with site occupancy factors 0.76 (2) and 0.24 (2) respectively; corresponding C atoms were restrained to have the same anisotropic displacement parameters.

### Crystal data

**Table:**

[Fe(C_5_H_5_)(C_14_H_10_N)]	*F*_000_ = 648
*M**_r_* = 313.17	*D*_x_ = 1.354 Mg m^−^^3^
Monoclinic, *P*2_1_/*c*	Mo *K*α radiation λ = 0.71073 Å
Hall symbol: -P 2ybc	Cell parameters from 2947 reflections
*a* = 6.8255 (14) Å	θ = 3.1–27.5º
*b* = 11.795 (2) Å	µ = 0.97 mm^−^^1^
*c* = 19.939 (4) Å	*T* = 293 (2) K
β = 106.786 (16)º	Block, red
*V* = 1536.8 (5) Å^3^	0.18 × 0.06 × 0.05 mm
*Z* = 4	

### Data collection

**Table:**

Rigaku, SCXmini diffractometer	3523 independent reflections
Radiation source: fine-focus sealed tube	2520 reflections with *I* > 2σ(*I*)
Monochromator: graphite	*R*_int_ = 0.050
Detector resolution: 13.6612 pixels mm^-1^	θ_max_ = 27.5º
*T* = 293(2) K	θ_min_ = 3.1º
ω scans	*h* = −8→8
Absorption correction: Multi-scan(CrystalClear; Rigaku, 2005)	*k* = −15→15
*T*_min_ = 0.892, *T*_max_ = 1.00	*l* = −25→25
15093 measured reflections	

### Refinement

**Table:**

Refinement on *F*^2^	Secondary atom site location: difference Fourier map
Least-squares matrix: full	Hydrogen site location: inferred from neighbouring sites
*R*[*F*^2^ > 2σ(*F*^2^)] = 0.053	H-atom parameters constrained
*wR*(*F*^2^) = 0.126	*w* = 1/[σ^2^(*F*_o_^2^) + (0.0496*P*)^2^ + 0.4337*P*] where *P* = (*F*_o_^2^ + 2*F*_c_^2^)/3
*S* = 1.07	(Δ/σ)_max_ < 0.001
3523 reflections	Δρ_max_ = 0.22 e Å^−^^3^
206 parameters	Δρ_min_ = −0.40 e Å^−^^3^
30 restraints	Extinction correction: none
Primary atom site location: structure-invariant direct methods	

### Special details

**Table:**

Geometry. All esds (except the esd in the dihedral angle between two l.s. planes) are estimated using the full covariance matrix. The cell esds are taken into account individually in the estimation of esds in distances, angles and torsion angles; correlations between esds in cell parameters are only used when they are defined by crystal symmetry. An approximate (isotropic) treatment of cell esds is used for estimating esds involving l.s. planes.
Refinement. Refinement of *F*^2^ against ALL reflections. The weighted *R*-factor *wR* and goodness of fit *S* are based on *F*^2^, conventional *R*-factors *R* are based on *F*, with *F* set to zero for negative *F*^2^. The threshold expression of *F*^2^ > σ(*F*^2^) is used only for calculating *R*-factors(gt) *etc*. and is not relevant to the choice of reflections for refinement. *R*-factors based on *F*^2^ are statistically about twice as large as those based on *F*, and *R*- factors based on ALL data will be even larger.

### Fractional atomic coordinates and isotropic or equivalent isotropic displacement parameters (Å^2^)

**Table:**

	*x*	*y*	*z*	*U*_iso_*/*U*_eq_	Occ. (<1)
Fe1	0.27058 (7)	0.42653 (4)	0.29905 (2)	0.06096 (17)	
N1	−0.4172 (4)	0.4888 (3)	0.12481 (18)	0.0911 (10)	
C1	0.1205 (4)	0.3903 (2)	0.19717 (14)	0.0537 (6)	
C2	0.2972 (5)	0.3198 (3)	0.22231 (17)	0.0701 (8)	
H2A	0.4135	0.3160	0.2029	0.084*	
C3	0.2759 (7)	0.2573 (3)	0.2800 (2)	0.0887 (11)	
H3A	0.3752	0.2028	0.3077	0.106*	
C4	0.0914 (7)	0.2867 (3)	0.2915 (2)	0.0922 (12)	
H4A	0.0394	0.2566	0.3289	0.111*	
C5	−0.0077 (5)	0.3694 (3)	0.24149 (17)	0.0714 (8)	
H5A	−0.1400	0.4052	0.2377	0.086*	
C6	0.2256 (9)	0.5813 (5)	0.3412 (3)	0.0813 (15)	0.764 (8)
H6A	0.0944	0.6208	0.3326	0.098*	0.764 (8)
C7	0.3062 (13)	0.4980 (6)	0.3956 (3)	0.105 (2)	0.764 (8)
H7A	0.2460	0.4729	0.4322	0.125*	0.764 (8)
C8	0.4978 (13)	0.4657 (7)	0.3864 (4)	0.113 (2)	0.764 (8)
H8A	0.5923	0.4107	0.4158	0.136*	0.764 (8)
C9	0.5340 (9)	0.5257 (7)	0.3301 (4)	0.0948 (17)	0.764 (8)
H9A	0.6548	0.5189	0.3133	0.114*	0.764 (8)
C10	0.3661 (11)	0.5945 (6)	0.3021 (4)	0.0794 (17)	0.764 (8)
H10A	0.3473	0.6441	0.2613	0.095*	0.764 (8)
C6'	0.270 (3)	0.5375 (19)	0.3729 (13)	0.0813 (15)	0.236 (8)
H6'A	0.1502	0.5559	0.3885	0.098*	0.236 (8)
C7'	0.421 (5)	0.452 (2)	0.4048 (12)	0.105 (2)	0.236 (8)
H7'A	0.4250	0.4022	0.4445	0.125*	0.236 (8)
C8'	0.562 (4)	0.457 (3)	0.3629 (14)	0.113 (2)	0.236 (8)
H8'A	0.6793	0.4061	0.3681	0.136*	0.236 (8)
C9'	0.488 (4)	0.534 (3)	0.3082 (11)	0.0948 (17)	0.236 (8)
H9'A	0.5544	0.5509	0.2718	0.114*	0.236 (8)
C10'	0.315 (4)	0.588 (3)	0.3162 (16)	0.0794 (17)	0.236 (8)
H10B	0.2395	0.6496	0.2872	0.095*	0.236 (8)
C11	0.1004 (4)	0.4723 (2)	0.14174 (13)	0.0480 (6)	
H11A	0.2197	0.4875	0.1297	0.058*	
C12	−0.0664 (4)	0.5297 (2)	0.10511 (13)	0.0452 (6)	
C13	−0.0704 (4)	0.6196 (2)	0.05293 (13)	0.0483 (6)	
C14	−0.2528 (5)	0.6672 (3)	0.01394 (16)	0.0699 (8)	
H14A	−0.3752	0.6405	0.0197	0.084*	
C15	−0.2574 (6)	0.7535 (3)	−0.03341 (19)	0.0869 (11)	
H15A	−0.3820	0.7844	−0.0589	0.104*	
C16	−0.0805 (7)	0.7933 (3)	−0.04285 (19)	0.0854 (11)	
H16A	−0.0838	0.8524	−0.0740	0.103*	
C17	0.1036 (6)	0.7462 (3)	−0.00626 (19)	0.0788 (10)	
H17A	0.2244	0.7721	−0.0136	0.095*	
C18	0.1093 (5)	0.6603 (3)	0.04146 (16)	0.0636 (7)	
H18A	0.2345	0.6293	0.0663	0.076*	
C19	−0.2607 (4)	0.5059 (2)	0.11687 (15)	0.0588 (7)	

### Atomic displacement parameters (Å^2^)

**Table:**

	*U*^11^	*U*^22^	*U*^33^	*U*^12^	*U*^13^	*U*^23^
Fe1	0.0733 (3)	0.0567 (3)	0.0496 (2)	−0.0014 (2)	0.0125 (2)	0.00379 (19)
N1	0.0504 (16)	0.114 (3)	0.110 (3)	−0.0086 (16)	0.0258 (16)	0.017 (2)
C1	0.0581 (16)	0.0510 (14)	0.0512 (15)	−0.0058 (13)	0.0147 (13)	−0.0008 (12)
C2	0.083 (2)	0.0592 (18)	0.0641 (18)	0.0146 (16)	0.0151 (16)	0.0034 (15)
C3	0.118 (3)	0.0534 (19)	0.082 (2)	0.005 (2)	0.009 (2)	0.0107 (17)
C4	0.115 (3)	0.081 (2)	0.077 (2)	−0.029 (2)	0.023 (2)	0.022 (2)
C5	0.0686 (19)	0.082 (2)	0.0657 (19)	−0.0166 (17)	0.0221 (16)	0.0088 (17)
C6	0.121 (4)	0.063 (3)	0.060 (3)	0.006 (3)	0.026 (3)	−0.005 (2)
C7	0.183 (7)	0.075 (4)	0.046 (3)	0.018 (4)	0.020 (4)	0.001 (2)
C8	0.133 (6)	0.107 (4)	0.068 (6)	0.021 (4)	−0.022 (3)	−0.021 (4)
C9	0.070 (4)	0.107 (4)	0.085 (5)	−0.015 (3)	−0.013 (3)	−0.019 (4)
C10	0.089 (5)	0.066 (2)	0.082 (4)	−0.019 (3)	0.023 (3)	−0.008 (2)
C6'	0.121 (4)	0.063 (3)	0.060 (3)	0.006 (3)	0.026 (3)	−0.005 (2)
C7'	0.183 (7)	0.075 (4)	0.046 (3)	0.018 (4)	0.020 (4)	0.001 (2)
C8'	0.133 (6)	0.107 (4)	0.068 (6)	0.021 (4)	−0.022 (3)	−0.021 (4)
C9'	0.070 (4)	0.107 (4)	0.085 (5)	−0.015 (3)	−0.013 (3)	−0.019 (4)
C10'	0.089 (5)	0.066 (2)	0.082 (4)	−0.019 (3)	0.023 (3)	−0.008 (2)
C11	0.0455 (14)	0.0553 (14)	0.0461 (14)	0.0003 (12)	0.0177 (11)	−0.0021 (12)
C12	0.0406 (13)	0.0525 (14)	0.0433 (13)	−0.0017 (11)	0.0135 (11)	−0.0073 (11)
C13	0.0522 (14)	0.0519 (14)	0.0394 (13)	0.0001 (12)	0.0108 (11)	−0.0072 (11)
C14	0.0583 (17)	0.088 (2)	0.0609 (18)	0.0090 (16)	0.0133 (15)	0.0151 (16)
C15	0.082 (2)	0.102 (3)	0.069 (2)	0.023 (2)	0.0105 (19)	0.025 (2)
C16	0.114 (3)	0.076 (2)	0.065 (2)	0.004 (2)	0.025 (2)	0.0182 (18)
C17	0.082 (2)	0.078 (2)	0.077 (2)	−0.0134 (19)	0.0245 (19)	0.0116 (18)
C18	0.0584 (17)	0.0674 (19)	0.0616 (18)	−0.0044 (15)	0.0119 (14)	0.0060 (15)
C19	0.0492 (16)	0.0637 (17)	0.0605 (17)	−0.0033 (14)	0.0112 (13)	0.0009 (14)

### Geometric parameters (Å, °)

**Table:**

Fe1—C9'	1.92 (3)	C8—H8A	0.9800
Fe1—C10'	1.94 (3)	C9—C10	1.383 (7)
Fe1—C6'	1.970 (19)	C9—H9A	0.9800
Fe1—C8	2.023 (7)	C10—H10A	0.9800
Fe1—C5	2.029 (3)	C6'—C10'	1.388 (17)
Fe1—C2	2.030 (3)	C6'—C7'	1.451 (18)
Fe1—C4	2.032 (4)	C6'—H6'A	0.9800
Fe1—C3	2.034 (4)	C7'—C8'	1.445 (19)
Fe1—C1	2.039 (3)	C7'—H7'A	0.9800
Fe1—C7	2.051 (5)	C8'—C9'	1.396 (18)
Fe1—C8'	2.06 (3)	C8'—H8'A	0.9800
Fe1—C6	2.069 (5)	C9'—C10'	1.393 (17)
N1—C19	1.142 (4)	C9'—H9'A	0.9800
C1—C2	1.431 (4)	C10'—H10B	0.9800
C1—C5	1.433 (4)	C11—C12	1.345 (3)
C1—C11	1.445 (4)	C11—H11A	0.9300
C2—C3	1.409 (5)	C12—C19	1.439 (4)
C2—H2A	0.9800	C12—C13	1.480 (4)
C3—C4	1.387 (5)	C13—C14	1.382 (4)
C3—H3A	0.9800	C13—C18	1.396 (4)
C4—C5	1.419 (5)	C14—C15	1.383 (5)
C4—H4A	0.9800	C14—H14A	0.9300
C5—H5A	0.9800	C15—C16	1.359 (5)
C6—C10	1.408 (8)	C15—H15A	0.9300
C6—C7	1.449 (7)	C16—C17	1.375 (5)
C6—H6A	0.9800	C16—H16A	0.9300
C7—C8	1.424 (10)	C17—C18	1.382 (4)
C7—H7A	0.9800	C17—H17A	0.9300
C8—C9	1.408 (10)	C18—H18A	0.9300
			
C9'—Fe1—C10'	42.3 (7)	C4—C5—Fe1	69.7 (2)
C9'—Fe1—C6'	70.2 (8)	C1—C5—Fe1	69.73 (17)
C10'—Fe1—C6'	41.5 (6)	C4—C5—H5A	126.3
C9'—Fe1—C8	52.3 (7)	C1—C5—H5A	126.3
C10'—Fe1—C8	65.3 (9)	Fe1—C5—H5A	126.3
C6'—Fe1—C8	51.1 (7)	C10—C6—C7	108.5 (5)
C9'—Fe1—C5	147.7 (7)	C10—C6—Fe1	70.6 (4)
C10'—Fe1—C5	119.9 (8)	C7—C6—Fe1	68.8 (3)
C6'—Fe1—C5	116.1 (6)	C10—C6—H6A	125.7
C8—Fe1—C5	157.2 (3)	C7—C6—H6A	125.7
C9'—Fe1—C2	104.1 (8)	Fe1—C6—H6A	125.7
C10'—Fe1—C2	134.2 (8)	C8—C7—C6	104.4 (6)
C6'—Fe1—C2	174.3 (6)	C8—C7—Fe1	68.5 (3)
C8—Fe1—C2	125.3 (3)	C6—C7—Fe1	70.1 (3)
C5—Fe1—C2	68.93 (14)	C8—C7—H7A	127.8
C9'—Fe1—C4	166.9 (9)	C6—C7—H7A	127.8
C10'—Fe1—C4	150.3 (8)	Fe1—C7—H7A	127.8
C6'—Fe1—C4	117.6 (7)	C9—C8—C7	110.3 (7)
C8—Fe1—C4	123.0 (3)	C9—C8—Fe1	72.3 (4)
C5—Fe1—C4	40.89 (14)	C7—C8—Fe1	70.6 (4)
C2—Fe1—C4	67.89 (17)	C9—C8—H8A	124.8
C9'—Fe1—C3	127.5 (9)	C7—C8—H8A	124.8
C10'—Fe1—C3	169.8 (8)	Fe1—C8—H8A	124.8
C6'—Fe1—C3	142.7 (8)	C10—C9—C8	107.5 (6)
C8—Fe1—C3	109.5 (3)	C10—C9—Fe1	70.5 (4)
C5—Fe1—C3	68.47 (16)	C8—C9—Fe1	67.7 (4)
C2—Fe1—C3	40.58 (14)	C10—C9—H9A	126.2
C4—Fe1—C3	39.89 (15)	C8—C9—H9A	126.2
C9'—Fe1—C1	112.6 (6)	Fe1—C9—H9A	126.2
C10'—Fe1—C1	112.9 (8)	C9—C10—C6	109.2 (5)
C6'—Fe1—C1	140.8 (8)	C9—C10—Fe1	70.7 (4)
C8—Fe1—C1	161.0 (3)	C6—C10—Fe1	69.7 (3)
C5—Fe1—C1	41.24 (12)	C9—C10—H10A	125.4
C2—Fe1—C1	41.19 (12)	C6—C10—H10A	125.4
C4—Fe1—C1	68.75 (13)	Fe1—C10—H10A	125.4
C3—Fe1—C1	68.87 (13)	C10'—C6'—C7'	110.9 (15)
C9'—Fe1—C7	76.2 (7)	C10'—C6'—Fe1	68.1 (16)
C10'—Fe1—C7	57.4 (8)	C7'—C6'—Fe1	73.2 (14)
C6'—Fe1—C7	18.4 (6)	C10'—C6'—H6'A	124.6
C8—Fe1—C7	40.9 (3)	C7'—C6'—H6'A	124.6
C5—Fe1—C7	120.3 (3)	Fe1—C6'—H6'A	124.6
C2—Fe1—C7	161.9 (3)	C8'—C7'—C6'	103.2 (14)
C4—Fe1—C7	108.0 (2)	C8'—C7'—Fe1	68.6 (16)
C3—Fe1—C7	125.1 (2)	C6'—C7'—Fe1	64.9 (13)
C1—Fe1—C7	155.6 (3)	C8'—C7'—H7'A	128.4
C9'—Fe1—C8'	40.9 (6)	C6'—C7'—H7'A	128.4
C10'—Fe1—C8'	69.4 (9)	Fe1—C7'—H7'A	128.4
C6'—Fe1—C8'	68.5 (8)	C9'—C8'—C7'	109.1 (16)
C8—Fe1—C8'	20.8 (6)	C9'—C8'—Fe1	64.2 (15)
C5—Fe1—C8'	170.4 (8)	C7'—C8'—Fe1	70.5 (16)
C2—Fe1—C8'	107.0 (7)	C9'—C8'—H8'A	125.2
C4—Fe1—C8'	129.7 (8)	C7'—C8'—H8'A	125.2
C3—Fe1—C8'	102.6 (8)	Fe1—C8'—H8'A	125.2
C1—Fe1—C8'	140.3 (7)	C10'—C9'—C8'	109.5 (16)
C7—Fe1—C8'	61.1 (7)	C10'—C9'—Fe1	69.7 (17)
C9'—Fe1—C6	65.3 (9)	C8'—C9'—Fe1	74.9 (16)
C10'—Fe1—C6	25.6 (7)	C10'—C9'—H9'A	125.2
C6'—Fe1—C6	22.8 (6)	C8'—C9'—H9'A	125.2
C8—Fe1—C6	67.4 (3)	Fe1—C9'—H9'A	125.2
C5—Fe1—C6	107.3 (2)	C6'—C10'—C9'	107.1 (15)
C2—Fe1—C6	155.4 (2)	C6'—C10'—Fe1	70.3 (14)
C4—Fe1—C6	126.1 (2)	C9'—C10'—Fe1	68.0 (17)
C3—Fe1—C6	162.6 (2)	C6'—C10'—H10B	126.5
C1—Fe1—C6	119.9 (2)	C9'—C10'—H10B	126.5
C7—Fe1—C6	41.2 (2)	Fe1—C10'—H10B	126.5
C8'—Fe1—C6	80.0 (7)	C12—C11—C1	129.1 (2)
C2—C1—C5	106.7 (3)	C12—C11—H11A	115.4
C2—C1—C11	122.8 (3)	C1—C11—H11A	115.4
C5—C1—C11	130.3 (3)	C11—C12—C19	119.3 (2)
C2—C1—Fe1	69.07 (17)	C11—C12—C13	125.7 (2)
C5—C1—Fe1	69.03 (17)	C19—C12—C13	115.0 (2)
C11—C1—Fe1	122.09 (19)	C14—C13—C18	117.4 (3)
C3—C2—C1	108.4 (3)	C14—C13—C12	121.1 (2)
C3—C2—Fe1	69.9 (2)	C18—C13—C12	121.5 (2)
C1—C2—Fe1	69.74 (17)	C13—C14—C15	121.5 (3)
C3—C2—H2A	125.8	C13—C14—H14A	119.3
C1—C2—H2A	125.8	C15—C14—H14A	119.3
Fe1—C2—H2A	125.8	C16—C15—C14	120.2 (3)
C4—C3—C2	108.4 (3)	C16—C15—H15A	119.9
C4—C3—Fe1	70.0 (2)	C14—C15—H15A	119.9
C2—C3—Fe1	69.55 (19)	C15—C16—C17	120.0 (3)
C4—C3—H3A	125.8	C15—C16—H16A	120.0
C2—C3—H3A	125.8	C17—C16—H16A	120.0
Fe1—C3—H3A	125.8	C16—C17—C18	120.1 (3)
C3—C4—C5	109.1 (3)	C16—C17—H17A	120.0
C3—C4—Fe1	70.1 (2)	C18—C17—H17A	120.0
C5—C4—Fe1	69.45 (19)	C17—C18—C13	120.9 (3)
C3—C4—H4A	125.4	C17—C18—H18A	119.6
C5—C4—H4A	125.4	C13—C18—H18A	119.6
Fe1—C4—H4A	125.4	N1—C19—C12	178.3 (3)
C4—C5—C1	107.4 (3)		
